# Supplementary material for: Using tree-based ensemble methods to produce a population-based mortality risk score in Ontario, Canada
Source: PLoS One. 2026 Apr 23;21(4):e0347302. doi: 10.1371/journal.pone.0347302 (PMC13105360; doi:10.1371/journal.pone.0347302)
Supplement: S1 File — International Journal of Medical Informatics (IJMEDI) checklist checklist for assessment of medical artificial intelligence. (PDF) [file pone.0347302.s001.pdf]

STROBE Statement—checklist of items that should be included in reports of observational studies

| | Item No. | Recommendation | Page No. | Relevant text from manuscript |
|---|---|---|---|---|
| **Title and abstract** | 1 | (*a*) Indicate the study's design with a commonly used term in the title or the abstract | 2 | This was a population-based retrospective cohort study. |
| | | (*b*) Provide in the abstract an informative and balanced summary of what was done and what was found | 2 | Methods and results section of abstract |
| **Introduction** | | | | |
| Background/rationale | 2 | Explain the scientific background and rationale for the investigation being reported | 3 | Quantifying the sickness of a population can be useful for health system planning. We explore a range of tree-based ensemble methods to predict the risk of 1-year mortality in a general adult population. |
| Objectives | 3 | State specific objectives, including any prespecified hypotheses | 3 | Purpose: In the present study, we construct models predicting all-cause 1-year mortality in the general adult population in Ontario, Canada. We uncover some of the black-box features of machine learning methods and provide additional exposure for epidemiologists and health system analysts interested in predictive modelling. |
| **Methods** | | | | |
| Study design | 4 | Present key elements of study design early in the paper | 4 | |
| Setting | 5 | Describe the setting, locations, and relevant dates, including periods of recruitment, exposure, follow-up, and data collection | 4-5 | All Ontarians alive as of January 1, 2022 were included. |

| | | | | |
|---|---|---|---|---|
| | | | | Lookback period of 1-3 years for risk factor assessment; look-forward period of 1 year for all-cause mortality. |
| Participants | 6 | (*a*) *Cohort study*—Give the eligibility criteria, and the sources and methods of selection of participants. Describe methods of follow-up<br>*Case-control study*—Give the eligibility criteria, and the sources and methods of case ascertainment and control selection. Give the rationale for the choice of cases and controls<br>*Cross-sectional study*—Give the eligibility criteria, and the sources and methods of selection of participants | 4 | Patient inclusion and exclusion criteria. |
| | | (*b*) *Cohort study*—For matched studies, give matching criteria and number of exposed and unexposed<br>*Case-control study*—For matched studies, give matching criteria and the number of controls per case | | n/a |
| Variables | 7 | Clearly define all outcomes, exposures, predictors, potential confounders, and effect modifiers. Give diagnostic criteria, if applicable | 4-5 | Covariates identified and measured |
| Data sources/ measurement | 8* | For each variable of interest, give sources of data and details of methods of assessment (measurement). Describe comparability of assessment methods if there is more than one group | 4-5 | |
| Bias | 9 | Describe any efforts to address potential sources of bias | n/a | |
| Study size | 10 | Explain how the study size was arrived at | 4 | Cohort creation subsection |

Continued on next page

| | | | | |
|---|---|---|---|---|
| Quantitative variables | 11 | Explain how quantitative variables were handled in the analyses. If applicable, describe which groupings were chosen and why | | |
| Statistical methods | 12 | (*a*) Describe all statistical methods, including those used to control for confounding | 6-12 | Subsections used to describe the 1) models; 2) assessing model performance; 3) sensitivity analyses; and 4) explainability |
| | | (*b*) Describe any methods used to examine subgroups and interactions | n/a | |
| | | (*c*) Explain how missing data were addressed | 4-5 | Analysis was performed on complete-case. |
| | | (*d*) *Cohort study*—If applicable, explain how loss to follow-up was addressed<br>*Case-control study*—If applicable, explain how matching of cases and controls was addressed<br>*Cross-sectional study*—If applicable, describe analytical methods taking account of sampling strategy | n/a | |
| | | (*e*) Describe any sensitivity analyses | 9-10 | Sensitivity analyses on 1) the definition of select comorbidities; 2) source of comorbidity; 3) data splitting; and 4) hyperparameter tuning |
| **Results** | | | | |
| Participants | 13* | (a) Report numbers of individuals at each stage of study—eg numbers potentially eligible, examined for eligibility, confirmed eligible, included in the study, completing follow-up, and analysed | 12 | |
| | | (b) Give reasons for non-participation at each stage | n/a | |
| | | (c) Consider use of a flow diagram | n/a | |
| Descriptive data | 14* | (a) Give characteristics of study participants (eg demographic, clinical, social) and information on exposures and potential confounders | Table 1 | |
| | | (b) Indicate number of participants with missing data for each variable of interest | n/a | |
| | | (c) *Cohort study*—Summarise follow-up time (eg, average and total amount) | n/a | |
| Outcome data | 15* | *Cohort study*—Report numbers of outcome events or summary measures over time | n/a | Temporal component n/a |
| | | *Case-control study*—Report numbers in each exposure category, or summary measures of exposure | n/a | |
| | | *Cross-sectional study*—Report numbers of outcome events or summary measures | n/a | |
| Main results | 16 | (*a*) Give unadjusted estimates and, if applicable, confounder-adjusted estimates and their precision | Table 1-2 | |

| | | | | |
|---|---|---|---|---|
| | | (eg, 95% confidence interval). Make clear which confounders were adjusted for and why they were included | | |
| | | (*b*) Report category boundaries when continuous variables were categorized | Table 1 and Table 3 | Table 1 for ADL<br>Table 3 for predicted probability cut-points (for visualization only) |
| | | (*c*) If relevant, consider translating estimates of relative risk into absolute risk for a meaningful time period | eTable S5 | Average and relative marginal effects |
| Other analyses | 17 | Report other analyses done—eg analyses of subgroups and interactions, and sensitivity analyses | 17-19 | Sensitivity analyses and explainability |
| **Discussion** | | | | |
| Key results | 18 | Summarise key results with reference to study objectives | 19 | |
| Limitations | 19 | Discuss limitations of the study, taking into account sources of potential bias or imprecision. Discuss both direction and magnitude of any potential bias | 22-23 | Limitations subsection of discussion |
| Interpretation | 20 | Give a cautious overall interpretation of results considering objectives, limitations, multiplicity of analyses, results from similar studies, and other relevant evidence | 24 | Conclusion subsection of discussion |
| Generalisability | 21 | Discuss the generalisability (external validity) of the study results | 23 | Importantly, we expect these findings to be generalizable to other jurisdictions because comorbidity indices have been demonstrated to be similarly prognostic internationally |
| **Other information** | | | | |
| Funding | 22 | Give the source of funding and the role of the funders for the present study and, if applicable, for the original study on which the present article is based | 1 | Funding: No specific sources of funding were used |

*Give information separately for cases and controls in case-control studies and, if applicable, for exposed and unexposed groups in cohort and cross-sectional studies.

**Note:** An Explanation and Elaboration article discusses each checklist item and gives methodological background and published examples of transparent reporting. The STROBE checklist is best used in conjunction with this article (freely available on the Web sites of PLoS Medicine at http://www.plosmedicine.org/, Annals of Internal Medicine at http://www.annals.org/, and Epidemiology at http://www.epidem.com/). Information on the STROBE Initiative is available at www.strobe-statement.org.

# The IJMEDI checklist for assessment of medical AI

| Requirement | Authors | | | Reviewers | | |
|---|---|---|---|---|---|---|
| | NA | No | Yes | OK | mR | MR |
| Problem Understanding | | | | | | |
| **1. Is the study population described, also in terms of inclusion/exclusion criteria (e.g., patients older than 18 tested for COVID-19; all inpatients hospitalized for 24 or more hours)? §** | ○ | ○ | ○ ✓ | ○ | ○ | ○ |
| **2. Is the study design described? (e.g., retrospective, prospective, cross-sectional [1], observational, randomized control trial [2]) §** | ○ | ○ | ○ ✓ | ○ | ○ | ○ |
| 3. Is the study setting described? (e.g., teaching tertiary hospital; primary care ambulatory, nursing home, medical laboratory, R&D laboratory) § | ○ | ○ | ○ ✓ | ○ | ○ | ○ |
| 4. **Is the source of data described? (e.g., electronic specialty registry; laboratory information system; electronic health record; picture archiving and communication system) §** | ○ | ○ | ○ ✓ | ○ | ○ | ○ |
| **5. Is the medical task reported? (e.g., diagnostic detection, diagnostic characterization, diagnostic staging, prognosis (on which endpoint), event prediction, risk stratification, anatomical structure segmentation, treatment selection and planning, monitoring)** § | ○ | ○ | ○ ✓ | ○ | ○ | ○ |
| 6. Is the data collection process described, also in terms of setting-specific data collection strategies (e.g. whether body temperatures are measured only in the morning; whether some blood tests are performed only in light of a specific diagnostic hypothesis)? Any consideration about data quality is appreciated, e.g., in regard to completeness, plausibility, and robustness with respect to upcoding or downcoding practices | ○ | ○ | ○ ✓ | ○ | ○ | ○ |
| Data Understanding | | | | | | |
| **7. Are the subject demographics described in terms of**<br>**1. average age (mean or median);**<br>**2. age variability (standard deviation (SD) or inter-quartile range (IQR));**<br>**3. gender breakdown (e.g., 55% female, 44% male, 1% not reported); §**<br>**4. main comorbidities;**<br>**5. ethnic group (e.g., Native American, Asian, South East Asian, African, African American, Hispanic, Native Hawaiian or Other Pacific Islander, European or American White);**<br>**6. socioeconomic status?** | ○ | ○ | ○ ✓ | ○ | ○ | ○ |
| **8. If the task is supervised, is the gold standard described? (e.g., "100 manually annotated clinical notes and pain scores recorded in EHR, Death, re-admission and International Classification of Disease (ICD) codes in discharge letters"). In particular, the authors should describe the process of ground truthing described in terms of:**<br>**1. Number of annotators (raters) producing the labels;**<br>**2. Their profession and expertise (e.g., years from specialization or graduation);**<br>**3. Particular instructions given to annotators for quality control (e.g., which data were discarded and why);**<br>**4. Inter-rater agreement score (e.g., Alpha [3], Kappa [4], Rho [5]);**<br>**5. Labelling technique (e.g., majority voting, Delphi method [6], consensus iteration).** | ○ | ○ | ○ ✓ | ○ | ○ | ○ |

| Requirement | Authors | | | Reviewers | | |
|---|---|---|---|---|---|---|
| | NA | No | Yes | OK | mR | MR |
| **9. In the case of tabular data, are the features described (also in regard to how they were used in the model in terms of categories or transformation)? This description should be done for all, or, in the case that the features exceed 20, for a significant subset of the most predictive features in the following terms: name, short description, type (nominal, ordinal, continuous), and**<br>**1. If continuous: unit of measure, range (min, max), mean and standard deviation (or median and IQR). Violin plots of some relevant continuous features are appreciated. If data are hematochemical parameters, also mention the brand and model of the analyzer equipment.**<br>**2. If nominal, all codes/values and their distribution. Feature transformation (e.g. one-hot encoding) should be reported if applied. Any terminology standard should be explicitly mentioned (e.g., LOINC [7], ICD-11 [8], SNOMED [9]) if applied.** | ○ | ○ | ✓ | ○ | ○ | ○ |
| Data Preparation | | | | | | |
| **10. Is outlier detection and analysis performed and reported? If the answer is yes, the definition of an outlier should be given and the techniques applied to manage outliers should be described (e.g., removal through application of an Isolation Forest model).** | ○ | ○ | ✓ | ○ | ○ | ○ |
| **11. Is missing-value management described? This description should be reported in the following terms:**<br>**1. The missing rate for each feature should be reported;**<br>**2. The technique of imputation, if any, should be described, and reasons for its choice should be given. If the missing rate is higher than 10%, a reflection about the impact on the performance of a technique with respect to others would be appreciable [10].** | ○ | ○ | ✓ | ○ | ○ | ○ |
| **12. Is feature pre-processing performed and described? This description should be reported in terms of scaling transformations (e.g. normalization, standardization, log-transformation) or discretization procedures applied to continuous features, and encoding of categorical or ordinal variables (e.g., one-hot encoding, ordinal encoding).** | ○ | ○ | ✓ | ○ | ○ | ○ |
| **13. Is data imbalance analysis and adjustment performed and reported? The authors should describe any imbalance in the data distribution, both in regard to the target (e.g. only 10% of the patients were affected by a given disease); and in regard to important predictive features (e.g. female patients accounted for less than 10% of the total cases). The authors should also report about any technique (if any) applied to adjust the above mentioned imbalances (e.g. under- or over-sampling, SMOTE).** | ○ | ○ | ✓ | ○ | ○ | ○ |
| Modeling | | | | | | |
| **14. Is the model task reported? (e.g., binary classification, multi-class classification, multi-label classification, ordinal regression, continuous regression, clustering, dimensionality reduction, segmentation) §** | ○ | ○ | ✓ | ○ | ○ | ○ |
| **15. Is the model output specified? (e.g., disease positivity probability score, probability of infection within 5 days, postoperative 3-month pain scores) §** | ○ | ○ | ✓ | ○ | ○ | ○ |
| **16. Is the model architecture or type described? (e.g., SVM, Random Forest, Boosting, Logistic Regression, Nearest Neighbors, Convolutional Neural Network)** | ○ | ○ | ✓ | ○ | ○ | ○ |
| Validation | | | | | | |
| **17. Is the data splitting [11] described (e.g., no data splitting;, k-fold cross-validation (CV); nested k-fold CV; repeated CV; bootstrap validation; leave-one-out CV; 80%/10%10% train/validation/test)? In the case of data splitting, the authors must explicitly state that splitting was performed before any pre-processing steps (e.g. normalization, standardization, missing value imputation, feature selection) or model construction steps (training, hyper-parameter optimization), so to avoid data leakage [12] and overfitting.** | ○ | ○ | ✓ | ○ | ○ | ○ |

| Requirement | Authors | | | Reviewers | | |
|---|---|---|---|---|---|---|
| | NA | No | Yes | OK | mR | MR |
| **18. Is the model training and selection described? In particular, the training procedure, hyper-parameter optimization or model selection should be described in terms of**<br>**1. Range of hyper-parameters [13];**<br>**2. Method used to select the best hyper-parameter configuration (e.g., Hyper-parameter selection was performed through nested k-fold CV based grid search);**<br>**3. Full specification of the hyper-parameters used to generate results [13];**<br>**4. Procedure (if any) to limit over-fitting, in particular as related to the sample size [14].** | ○ | ○ | ✓ | ○ | ○ | ○ |
| **19. (classification models) Is the model calibration described? If the answer is yes, the Brier score should be reported, and a calibration plot should be presented [15]** | ○ | ○ | ✓ | ○ | ○ | ○ |
| **20. Is the internal/internal-external model validation procedure described [11, 16] (e.g., internal 10-fold CV, time-based cross-validation)? The authors should explicitly specify that the sets have been splitted before normalization, standardization and imputation, to avoid data leakage [12] (also refer to item 17 of this guideline). If possible, the authors should also comment on the adequacy of the available sample size for model training and validation [17, 14]. Moreover, the authors should try to choose the test set so that it is the most diverse with respect to the remainder of the sample [18] (w.r.t. some multivariate similarity function) and how this choice relates to conservative (and lower-bound) estimates of the model's accuracy (and performance)** | ○ | ○ | ✓ | ○ | ○ | ○ |
| 21. Has the model been externally validated [19]? If the answer is yes, the characteristics of the external validation set(s) should be described. For instance, the authors could comment about the heterogeneity of the data with respect to the training set (e.g., degree of correspondence $\Psi$ [18], Data Representativeness Criterion [20]) and the cardinality of the external sample [21]. If the performance on external datasets is found to be comparable with (or better than) that on training and internal datasets, the authors should provide some explanatory conjectures for why this happened (e.g., high heterogeneity of the training set, high homogeneity of the external dataset) | ○ | ✓ | ○ | ○ | ○ | ○ |

| Requirement | Authors | | | Reviewers | | |
|---|---|---|---|---|---|---|
| | NA | No | Yes | OK | mR | MR |
| **22. Are the main error-based metrics used?**<br><br>**1. a. Classification performance should be reported in terms of: Accuracy, Balanced accuracy, Specificity, Sensitivity (recall), Area Under the Curve (if the positive condition is extremely rare - as in case of stroke events - authors could consider the "Area under the Precision-Recall Curve" [22]). Optionally also in terms of: positive and negative predictive value, F1 score, Matthew coefficient [23], F score of sensitivity and specificity, the full confusion matrix, Hamming Loss (for multi-label classification), Jaccard Index (for multi-label classification).**<br>**2. Regression performance should be reported in terms of: $R^2$; Mean Absolute Error (MAE); Root Mean Square Error (RMSE); Ratio between MAE (or RMSE) and SD (of the target)**<br>**3. Clustering performance should be reported in terms of: External validation metrics (e.g. mutual information, purity, Rand index), when ground truth labels are available, and Internal validation metrics (e.g. Davies-Bouldin index, Silhouette index, Homogeneity). The reported results of internal validation metrics should be discussed [24]**<br>**4. Image segmentation performance, depending on the specific task, should be reported in terms of metrics like [25]: accuracy-based metrics (e.g. Pixel accuracy, Jaccard Index, Dice Coefficient), distance-based metrics (e.g. mean absolute, or maximum difference), or area-based metrics (e.g. true positive fraction, true negative fraction, false positive fraction, false negative fraction).**<br>**5. Reinforcement learning performance, depending on the specific task, should be reported in terms of metrics like [26]: Fixed-Policy Regret, Dispersion across Time, Dispersion across Runs, Risk across Time, Risk across Runs, Dispersion across Fixed-Policy Rollouts, Risk across Fixed-Policy Rollouts.**<br><br>**The above estimates should be expressed, whenever possible, with their 95% (or 90%) confidence intervals (CI), or with other indicators of variability [27], with respect to the evaluation metrics reported. In this case, the authors should report which methods were applied for the computation of the confidence intervals (e.g. whether k-fold CV or bootstrap was applied, normal approximation). When comparing multiple models, the authors should discuss the statistical significance of the observed differences [28] (e.g. through CI comparisons, or hypothesis testing). When comparing multiple regression models, a Taylor diagram [29] could be reported and discussed.** | ○ | ○ | ○ ✓ | ○ | ○ | ○ |
| 23. Are some relevant errors described? The authors should describe the characteristic of some noteworthy classification errors or cases for which the regression prediction was much higher ($> 2x$) than the MAE. If these cases represent statistical outliers for some covariates, the authors should comment on that. To detect relevant cases, the authors could focus on those cases on which the inter-rater agreement (either re ground truth or by comparing human vs. model's performance) is lowest. | ○ | ○ | ○ ✓ | ○ | ○ | ○ |
| Deployment | | | | | | |
| 24. Is the target user indicated? (e.g., clinician, radiologist, hospital management team, insurance company, patients) § | ○ | ○ | ○ ✓ | ○ | ○ | ○ |
| 25. (classification models) Is the utility of the model discussed? The authors should report the performance of a baseline model (e.g., logistic regression, Naive Bayes). Additionally, the authors could report the Net Benefit [30] or similar metrics and present utility curves [31]. In particular, the authors are encouraged to discuss the selection of appropriate risk thresholds [32]; the relative value of benefits (true positives/negatives) and harms (false positives/negatives); and the clinical utility of the proposed models [14]. | ○ | ○ | ○ ✓ | ○ | ○ | ○ |

| Requirement | Authors | | | Reviewers | | |
|---|---|---|---|---|---|---|
| | NA | No | Yes | OK | mR | MR |
| 26. Is information regarding model interpretability and explainability available [33] (e.g. feature importance, interpretable surrogate models, information about the model parameters)? Claims of "high" or "adequate" model interpretability (e.g., by means of visual aids like decision trees, Variable Importance Plots or Shapley Additive Exlanations Plots (SHAP) [34]) or model causability [35] should always be supported by some user study, even qualitative or questionnaire-based [36]. In the case surrogate models were applied, the authors should report about their fidelity [37, 38] | ○ | ○ | ✓ | ○ | ○ | ○ |
| 27. Is there any discussion regarding model fairness, ethical concerns or risks of bias [14, 39] (for a list of clinically relevant biases, refer to [40] )? If possible, the authors should report the model performance stratified for particularly relevant population strata [41] (e.g. model performance on male vs female subjects, or on minority groups) | ○ | ○ | ✓ | ○ | ○ | ○ |
| 28. Is any point made about the environmental sustainability of the model, or about the carbon footprint [42], of either the training phase or inference phase (use) of the model? If the answer is yes, then such a footprint should be expressed in terms of carbon dioxide equivalent ($CO_2$eq) and details about the estimation method should be given. Any efforts to this end will be appreciated, including those based on tools available online[1], as well as any attempts to popularise this concept, e.g. through equivalences with the consumption of everyday devices such as smartphones or kilometres travelled by a fossil-fuelled car[2] | ○ | ✓ | ○ | ○ | ○ | ○ |
| **29. Is code and data shared with the community [13, 43]? § If not, are reasons given? If code and data are shared, institutional repositories such as Zenodo should be preferred to private-owned repositories (arxiv, GitHub). If code is shared, specification of dependencies should be reported and a clear distinction between training code and evaluation code should be made. The authors should also state whether the developed system, either as a sand-box or as fully-operating system, has been made freely accessible on the Web.** | ○ | ○ | ✓ | ○ | ○ | ○ |
| 30. Is the system already adopted in daily practice? If the answer is *yes*, the authors should report on where (setting name) and since when. Moreover, appreciated additions would regard: the description on the digitized workflow integrating the system; any comment about the level of use [14]; a qualitative assessment of the level of efficacy of the system's contribution to the clinical process (e.g., [44, 45]); any comment about the technical and staff training effort actually required [14]. If the answer is *no*, the authors should be explicit in regard to the point in the clinical workflow where the ML model should be applied, possibly using standard notation (e.g., BPMN). Moreover, the authors should also propose an assessment of the technology readiness of the described system, with explicit reference to the Technology Readiness Level framework[3] or to any adaptation of this framework to the AI/ML domain [46]. In either above cases (yes/no), the authors should report about the procedures (if any) for performance monitoring, model maintenance and updating [47]. | ○ | ✓ | ○ | ○ | ○ | ○ |

Table 1: Checklist for assessment of requirements and recommendations for sound medical ML contributions to the existing literature. NA: not applicable; mR: minor revisions needed; MR: major revisions needed. Items in bold indicate priority aspects to be considered. Items denoted with a § symbol are directly inspired by the MINIMAR guideline [48]. The section names for the checklist items are directly inspired by the CRISP-DM framework [49].

[1] https://mlco2.github.io/impact/

[2] https://www.epa.gov/energy/greenhouse-gas-equivalencies-calculator

[3] Technology readiness levels (TRL) - Extract from Part 19 - Commission Decision C (2014) 4995

If you want to cite this checklist: Cabitza F., Campagner, A. (2021) The need to separate the wheat from the chaff in medical informatics. International Journal of Medical Informatics.

This work is licensed under a Creative Commons "Attribution-ShareAlike 4.0 International" license.

## References

[1] J. I. Hudson, H. G. Pope Jr, R. J. Glynn, The cross-sectional cohort study: an underutilized design, Epidemiology 16 (3) (2005) 355–359.

[2] E. L. Hannan, Randomized clinical trials and observational studies: guidelines for assessing respective strengths and limitations, JACC: Cardiovascular Interventions 1 (3) (2008) 211–217.

[3] K. Krippendorff, Content analysis: An introduction to its methodology, Sage publications, 2018.

[4] J. L. Fleiss, Measuring nominal scale agreement among many raters., Psychological bulletin 76 (5) (1971) 378.

[5] F. Cabitza, A. Campagner, D. Albano, A. Aliprandi, A. Bruno, V. Chianca, A. Corazza, F. Di Pietto, A. Gambino, S. Gitto, et al., The elephant in the machine: Proposing a new metric of data reliability and its application to a medical case to assess classification reliability, Applied Sciences 10 (11) (2020) 4014.

[6] H. A. Linstone, M. Turoff, et al., The delphi method, Addison-Wesley Reading, MA, 1975.

[7] C. J. McDonald, S. M. Huff, J. G. Suico, G. Hill, D. Leavelle, R. Aller, A. Forrey, K. Mercer, G. DeMoor, J. Hook, et al., Loinc, a universal standard for identifying laboratory observations: a 5-year update, Clinical chemistry 49 (4) (2003) 624–633.

[8] R.-D. Treede, W. Rief, A. Barke, Q. Aziz, M. I. Bennett, R. Benoliel, M. Cohen, S. Evers, N. B. Finnerup, M. B. First, et al., A classification of chronic pain for icd-11, Pain 156 (6) (2015) 1003.

[9] R. Cornet, N. de Keizer, Forty years of snomed: a literature review, BMC medical informatics and decision making 8 (1) (2008) 1–6.

[10] A. K. Waljee, A. Mukherjee, A. G. Singal, Y. Zhang, J. Warren, U. Balis, J. Marrero, J. Zhu, P. D. Higgins, Comparison of imputation methods for missing laboratory data in medicine, BMJ open 3 (8) (2013).

[11] S. Borra, A. Di Ciaccio, Measuring the prediction error. a comparison of cross-validation, bootstrap and covariance penalty methods, Computational statistics & data analysis 54 (12) (2010) 2976–2989.

[12] S. Kaufman, S. Rosset, C. Perlich, O. Stitelman, Leakage in data mining: Formulation, detection, and avoidance, ACM Transactions on Knowledge Discovery from Data (TKDD) 6 (4) (2012) 1–21.

[13] J. Pineau, P. Vincent-Lamarre, K. Sinha, V. Larivière, A. Beygelzimer, F. d'Alché Buc, E. Fox, H. Larochelle, Improving reproducibility in machine learning research (a report from the neurips 2019 reproducibility program), arXiv preprint arXiv:2003.12206 (2020).

[14] I. Scott, S. Carter, E. Coiera, Clinician checklist for assessing suitability of machine learning applications in healthcare, BMJ Health & Care Informatics 28 (1) (2021).

[15] B. Van Calster, D. J. McLernon, M. Van Smeden, L. Wynants, E. W. Steyerberg, Calibration: the achilles heel of predictive analytics, BMC medicine 17 (1) (2019) 1–7.

[16] E. W. Steyerberg, F. E. Harrell Jr, Prediction models need appropriate internal, internal-external, and external validation, Journal of clinical epidemiology 69 (2016) 245.

[17] I. Balki, A. Amirabadi, J. Levman, A. L. Martel, Z. Emersic, B. Meden, A. Garcia-Pedrero, S. C. Ramirez, D. Kong, A. R. Moody, et al., Sample-size determination methodologies for machine learning in medical imaging research: a systematic review, Canadian Association of Radiologists Journal 70 (4) (2019) 344–353.

[18] F. Cabitza, A. Campagner, L. M. Sconfienza, As if sand were stone. new concepts and metrics to probe the ground on which to build trustable ai, BMC Medical Informatics and Decision Making 20 (1) (2020) 1–21.

[19] S. Bleeker, H. Moll, E. Steyerberg, A. Donders, G. Derksen-Lubsen, D. Grobbee, K. Moons, External validation is necessary in prediction research: A clinical example, Journal of clinical epidemiology 56 (9) (2003) 826–832.

[20] E. Schat, R. van de Schoot, W. M. Kouw, D. Veen, A. M. Mendrik, The data representativeness criterion: Predicting the performance of supervised classification based on data set similarity, Plos one 15 (8) (2020) e0237009.

[21] K. I. Snell, L. Archer, J. Ensor, L. J. Bonnett, T. P. Debray, B. Phillips, G. S. Collins, R. D. Riley, External validation of clinical prediction models: simulation-based sample size calculations were more reliable than rules-of-thumb, Journal of clinical epidemiology 135 (2021) 79–89.

[22] B. Ozenne, F. Subtil, D. Maucort-Boulch, The precision–recall curve overcame the optimism of the receiver operating characteristic curve in rare diseases, Journal of clinical epidemiology 68 (8) (2015) 855–859.

[23] D. Chicco, N. Tötsch, G. Jurman, The matthews correlation coefficient (mcc) is more reliable than balanced accuracy, bookmaker informedness, and markedness in two-class confusion matrix evaluation, BioData mining 14 (1) (2021) 1–22.

[24] E. Rendón, I. Abundez, A. Arizmendi, E. M. Quiroz, Internal versus external cluster validation indexes, International Journal of computers and communications 5 (1) (2011) 27–34.

[25] A. Fenster, B. Chiu, Evaluation of segmentation algorithms for medical imaging, in: 2005 IEEE Engineering in Medicine and Biology 27th Annual Conference, IEEE, 2006, pp. 7186–7189.

[26] S. C. Chan, S. Fishman, J. Canny, A. Korattikara, S. Guadarrama, Measuring the reliability of reinforcement learning algorithms, in: International Conference on Learning Representations, Addis Ababa, Ethiopia, 2020.

[27] X. Bouthillier, P. Delaunay, M. Bronzi, A. Trofimov, B. Nichyporuk, J. Szeto, N. Mohammadi Sepahvand, E. Raff, K. Madan, V. Voleti, et al., Accounting for variance in machine learning benchmarks, Proceedings of Machine Learning and Systems 3 (2021).

[28] J. Demšar, Statistical comparisons of classifiers over multiple data sets, The Journal of Machine Learning Research 7 (2006) 1–30.

[29] K. E. Taylor, Summarizing multiple aspects of model performance in a single diagram, Journal of Geophysical Research: Atmospheres 106 (D7) (2001) 7183–7192.

[30] A. J. Vickers, B. Van Calster, E. W. Steyerberg, Net benefit approaches to the evaluation of prediction models, molecular markers, and diagnostic tests, bmj 352 (2016).

[31] B. Van Calster, L. Wynants, J. F. Verbeek, J. Y. Verbakel, E. Christodoulou, A. J. Vickers, M. J. Roobol, E. W. Steyerberg, Reporting and interpreting decision curve analysis: a guide for investigators, European urology 74 (6) (2018) 796–804.

[32] L. Wynants, M. van Smeden, D. J. McLernon, D. Timmerman, E. W. Steyerberg, B. Van Calster, Three myths about risk thresholds for prediction models, BMC medicine 17 (1) (2019) 1–7.

[33] A. Vellido, The importance of interpretability and visualization in machine learning for applications in medicine and health care, Neural computing and applications (2019) 1–15.

[34] M. Sundararajan, A. Najmi, The many shapley values for model explanation, in: International Conference on Machine Learning, PMLR, 2020, pp. 9269–9278.

[35] A. Holzinger, G. Langs, H. Denk, K. Zatloukal, H. Müller, Causability and explainability of artificial intelligence in medicine, Wiley Interdisciplinary Reviews: Data Mining and Knowledge Discovery 9 (4) (2019) e1312.

[36] A. Holzinger, A. Carrington, H. Müller, Measuring the quality of explanations: the system causability scale (scs), KI-Künstliche Intelligenz (2020) 1–6.

[37] R. R. Hoffman, S. T. Mueller, G. Klein, J. Litman, Metrics for explainable ai: Challenges and prospects, arXiv preprint arXiv:1812.04608 (2018).

[38] C. Schwartzenberg, T. van Engers, Y. Li, The fidelity of global surrogates in interpretable machine learning, BNAIC/BeneLearn 2020 (2020) 269.

[39] E. Vayena, A. Blasimme, I. G. Cohen, Machine learning in medicine: addressing ethical challenges, PLoS medicine 15 (11) (2018) e1002689.

[40] A. Rajkomar, M. Hardt, M. D. Howell, G. Corrado, M. H. Chin, Ensuring fairness in machine learning to advance health equity, Annals of internal medicine 169 (12) (2018) 866–872.

[41] L. Oakden-Rayner, J. Dunnmon, G. Carneiro, C. Ré, Hidden stratification causes clinically meaningful failures in machine learning for medical imaging, in: Proceedings of the ACM conference on health, inference, and learning, 2020, pp. 151–159.

[42] J. Cowls, A. Tsamados, M. Taddeo, L. Floridi, The ai gambit—leveraging artificial intelligence to combat climate change: Opportunities, challenges, and recommendations, https://papers.ssrn.com/sol3/papers.cfm?abstract_id=3804983 (2021).

[43] B. Van Calster, L. Wynants, D. Timmerman, E. W. Steyerberg, G. S. Collins, Predictive analytics in health care: how can we know it works?, Journal of the American Medical Informatics Association 26 (12) (2019) 1651–1654.

[44] D. G. Fryback, J. R. Thornbury, The efficacy of diagnostic imaging, Medical decision making 11 (2) (1991) 88–94.

[45] K. G. van Leeuwen, S. Schalekamp, M. J. Rutten, B. van Ginneken, M. de Rooij, Artificial intelligence in radiology: 100 commercially available products and their scientific evidence, European Radiology (2021) 1–8.

[46] A. Lavin, C. M. Gilligan-Lee, A. Visnjic, S. Ganju, D. Newman, S. Ganguly, D. Lange, A. G. Baydin, A. Sharma, A. Gibson, et al., Technology readiness levels for machine learning systems, arXiv preprint arXiv:2101.03989 (2021).

[47] S. E. Davis, R. A. Greevy, T. A. Lasko, C. G. Walsh, M. E. Matheny, Comparison of prediction model performance updating protocols: Using a data-driven testing procedure to guide updating, in: AMIA Annual Symposium Proceedings, Vol. 2019, American Medical Informatics Association, 2019, p. 1002.

[48] T. Hernandez-Boussard, S. Bozkurt, J. P. Ioannidis, N. H. Shah, Minimar (minimum information for medical ai reporting): developing reporting standards for artificial intelligence in health care, Journal of the American Medical Informatics Association 27 (12) (2020) 2011–2015.

[49] R. Wirth, J. Hipp, Crisp-dm: Towards a standard process model for data mining, in: Proceedings of the 4th international conference on the practical applications of knowledge discovery and data mining, Vol. 1, Springer-Verlag London, UK, 2000.
