# Supplementary material for: Using tree-based ensemble methods to produce a population-based mortality risk score in Ontario, Canada
Source: PLoS One. 2026 Apr 23;21(4):e0347302. doi: 10.1371/journal.pone.0347302 (PMC13105360; doi:10.1371/journal.pone.0347302)
Supplement: S2 File — (DOCX) [file pone.0347302.s002.docx]

# Appendix 1: International Classification of Diseases (ICD) 10^th^ edition codes for comorbidity

| Comorbidity | Original weight | Updated weight | ICD-10 codes |
| --- | --- | --- | --- |
| Myocardial infarction (MI) | 1 | 1 | 'I21' 'I22' 'I252' |
| Congestive heart failure (CHF) | 1 | 1 | 'I43' 'I50' 'I099' 'I110' 'I130' 'I132' 'I255' 'I420' 'I425' 'I426' 'I427' 'I428' 'I429' 'P290' |
| Peripheral vascular disease (PVD) | 1 | 1 | 'I70' 'I710' 'I711' 'I712' 'I713' 'I714' 'I715' 'I716' 'I717' 'I718' 'I719' 'I731' 'I738' 'I739' 'I771' 'I790' 'I792' 'K551' 'K558' 'K559' 'Z958' 'Z959' |
| Cerebrovascular disease (CVD) | 1 | 1 (if no hemiplegia) | 'G45' 'G46' 'I60' 'I61' 'I62' 'I63' 'I64' 'I65' 'I66' 'I67' 'I68' 'I69' 'H340' |
| Dementia | 1 | 1 | 'F00' 'F01' 'F02' 'F03' 'G30' 'F051' 'G311' |
| Chronic pulmonary disease (CPD) | 1 | 1 | 'J40' 'J41' 'J42' 'J43' 'J44' 'J45' 'J46' 'J47' 'J60' 'J61' 'J62' 'J63' 'J64' 'J65' 'J66' 'J67' 'I278' 'I279' 'J684' 'J701' 'J703' |
| Connective tissue/rheumatoid disease (CTDRD) | 1 | 1 | 'M32' 'M33' 'M34' 'M315' 'M351' 'M353' 'M360' 'M05' 'M06' |
| Peptic ulcer disease (PUD) | 1 | 1 | 'K25' 'K26' 'K27' 'K28' |
| Mild liver disease (MLD) | 1 | 1 | 'B18' 'K73' 'K74' 'K700' 'K701' 'K702' 'K703' 'K709' 'K717' 'K713' 'K714' 'K715' 'K760' 'K762' 'K763' 'K764' 'K768' 'K769' 'Z944' |
| Diabetes without complications (DWOC) | 1 | 1 | 'E100' 'E101' 'E106' 'E108' 'E109' 'E110' 'E111' 'E116' 'E118' 'E119' 'E120' 'E121' 'E126' 'E128' 'E129' 'E130' 'E131' 'E136' 'E138' 'E139' 'E140' 'E141' 'E146' 'E148' 'E149' |
| Diabetes with complications (DWC) | 2 | 2 | 'E102' 'E103' 'E104' 'E105' 'E107' 'E112' 'E113' 'E114' 'E115' 'E117' 'E122' 'E123' 'E124' 'E125' 'E127' 'E132' 'E133' 'E134' 'E135' 'E137' 'E142' 'E143' 'E144' 'E145' 'E147' |
| Hemiplegia/paraplegia | 2 | 2 | 'G81' 'G82' 'G041' 'G114' 'G801' 'G802' 'G830' 'G831' 'G832' 'G833' 'G834' 'G839' |
| Moderate-to-severe liver disease (MSLD) | 3 | 3 | 'K704' 'K711' 'K721' 'K729' 'K765' 'K766' 'K767' 'I850' 'I859' 'I864' 'I982' |
| Renal disease | 2 | n/a | 'N18' 'N19' 'N052' 'N053' 'N054' 'N055' 'N056' 'N057' 'N250' 'I120' 'I131' 'N032' 'N033' 'N034' 'N035' 'N036' 'N037' 'Z490' 'Z491' 'Z492' 'Z940' 'Z992' |
| Mild-to-moderate renal disease (MMRD) | n/a | 1 | 'I129' 'I130' 'I1310' 'N03' 'N05' 'N181' 'N182' 'N183' 'N184' 'N189' 'Z940' |
| Severe renal disease/end-stage renal disease | n/a | 3 | 'I120' 'I1311' 'I132' 'N185' 'N186' 'N19' 'N250' 'Z49' 'Z992' |
| Nephritic syndrome | n/a | n/a | 'N03' 'N05' |
| HIV infection | 6 | 3 | 'B20' 'B21' 'B22' 'B23' 'B24' |
| AIDS | n/a | 6 | HIV infection +  Opportunistic infection: 'B37' 'C53' 'B38' 'B45' 'A072' 'B25' 'G934' 'B00' 'B39' 'A073' 'C46' 'C81' 'C82' 'C83' 'C84' 'C85' 'C86' 'C87' 'C88' 'C89' 'C90' 'C91' 'C92' 'C93' 'C94' 'C95' 'C96' 'A31' 'A15' 'A16' 'A17' 'A18' 'A19' 'B59' 'Z8701' 'A812' 'A021' 'B58' 'R64' |
| Cancer (primary) | 2 | 2 | 'C00' 'C01' 'C02' 'C03' 'C04' 'C05' 'C06' 'C07' 'C08' 'C09' 'C10' 'C11' 'C12' 'C13' 'C14' 'C15' 'C16' 'C17' 'C18' 'C19' 'C20' 'C21' 'C22' 'C23' 'C24' 'C25' 'C26' 'C30' 'C31' 'C32' 'C33' 'C34' 'C37' 'C38' 'C39' 'C40' 'C41' 'C43' 'C45' 'C46' 'C47' 'C48' 'C49' 'C50' 'C51' 'C52' 'C53' 'C54' 'C55' 'C56' 'C57' 'C58' 'C60' 'C61' 'C62' 'C63' 'C64' 'C65' 'C66' 'C67' 'C68' 'C69' 'C70' 'C71' 'C72' 'C73' 'C74' 'C75' 'C76' 'C81' 'C82' 'C83' 'C84' 'C85' 'C88' 'C90' 'C91' 'C92' 'C93' 'C94' 'C95' 'C96' 'C97' |
| Cancer (metastatic) | 6 | 6 | 'C77' 'C78' 'C79' 'C80' |

Original weights were derived from Charlson (1987); updated weights are derived from Glasheen et al (2019).

# Appendix 2: Additional measures of health and healthcare utilization

| **Condition** | **Codes** | **Source** |
| --- | --- | --- |
| Long-term care | "W^^^" | OHIP |
| House call | 'A900' | OHIP |
| Mental health | 'K005' 'K007' 'K187' 'K188' 'K189' 'K198' 'K199' 'K197' 'K190' 'K192' 'G478' 'G479' | OHIP |
| American Society of Anesthesiologists (ASA) physical status classification |  |  |
| ASA V (moribund patient not expected to live 24 hours with or without operation) | 'E016' | OHIP |
| ASA IV (patient with incapacitating systemic disease that is a constant threat to life) | 'E017' | OHIP |
| ASA III (patient with severe systemic disease limiting activity but not incapacitating) | 'E022' | OHIP |
|  |  |  |
| Chronic pain | 'K707' | OHIP |
| Fibromyalgia | 'K037' | OHIP |
| Palliative care | 'K023' 'A945' 'C945' 'C982' 'G511' 'G512' | OHIP |
| Smoking cessation | 'K039' 'E079' | OHIP |
| Home care | 'K070' 'K071' 'K072' | OHIP |
| Substance abuse | 'A680' 'C680' 'K680' 'A957' 'G185' 'G200' | OHIP |
|  |  |  |
| Critical care use | 'G521' 'G522' 'G523' 'G391' 'G395' 'G391' 'G211' 'G400' 'G401' 'G402' 'G405' 'G406' 'G407' 'G557' 'G558' 'G559' 'G404' | OHIP |
| Hyperbaric treatment | 'G800' 'G801' 'G802' 'G804' 'G805' 'G807' | OHIP |
| Delivered a stillborn outcome | 'P006' 'P018' 'P020' 'P041' 'P042' | OHIP |
| Major burn | 'R039' 'Z180' 'Z181' 'R693' | OHIP |
| Amputation of upper or lower extremity (more than finger or toe) | 'R605' 'R613' 'R614' 'R617' 'R616' 'R615' 'R631' 'R630' 'R625' 'R626' 'R624' 'R620' 'R621' 'R623' 'R622' 'R619' 'R611' 'R612' | OHIP |
| Obesity | 'E010' 'E676' | OHIP |
|  |  |  |
| Hip fracture | 'M80' 'S72' and not a prosthesis 'T8403' | DAD |
| Delirium | F05* | DAD |
| Pressure injury (hospital-acquired) | L89* with diagnosis type = 2 | DAD |
|  |  |  |
| Vaginal delivery (CCI code) | 5MD5* | DAD |
| Caesarean delivery (CCI code) | 5MD60* | DAD |
| Influenza vaccine | 'G590' 'G591' 'G592' | OHIP |
| Breast cancer screening (3 years look-back) | 'X172' 'X178' | OHIP |
| Lookback period was 1-year unless otherwise stated.  CCI – Canadian Classification of Interventions (procedure codes)  DAD – Discharge Abstract Database  OHIP – Ontario Health Insurance Program. | | |

# Appendix 3: Defining a function to calculate the Integrated Calibration Index using quantiles

**#Calibration index equivalent - using centiles**

def calculate_ici_equivalent(y_pred_prob_test, y_test, num_bins = 100):

**# Create a histogram and extract bin edges**

counts, bin_edges = np.histogram(y_pred_prob_test, bins=num_bins) # 10 bins

mean_outcome = [] # Initialize an empty list to store mean outcomes

mean_prediction = [] # Initialize an empty list to store mean predictions

n = []

numerator = []

**#This code will ensure all bins have at least 1 observation. Otherwise we run into issues (mean_outcome is not defined)**

valid_bin_edges = [bin_edges[0]] # Start with the first edge

valid_counts = [] # To store counts of combined bins

for i in range(len(counts)):

if counts[i] == 0: # If the bin count is zero, skip adding a new bin edge

continue

valid_bin_edges.append(bin_edges[i + 1]) # Add the next bin edge

valid_counts.append(counts[i]) # Add the count for this bin

valid_bin_edges[-1] = 1.0001 #becasue there's no next bin to combine the previous one with

**# Iterate through bins**

for i in range(len(valid_counts)):

# Calculate conditional mean outcome and predicted probabilities

mean_outcome.append(np.mean(y_test[(y_pred_prob_test >= valid_bin_edges[i]) & (y_pred_prob_test < valid_bin_edges[i + 1])]))

mean_prediction.append(np.mean(y_pred_prob_test[(y_pred_prob_test >= valid_bin_edges[i]) & (y_pred_prob_test < valid_bin_edges[i + 1])]))

n.append(np.sum((y_pred_prob_test >= valid_bin_edges[i]) & (y_pred_prob_test < valid_bin_edges[i + 1])))

for i in range(len(n)):

numerator.append(np.sum(np.abs(mean_outcome[i]-mean_prediction[i])*n[i]))

**# Check results and some optional data quality checks**

#print("n:", n)

#print("Mean outcome:", mean_outcome)

#print("Mean predicted probabilities:", mean_prediction)

#should all be the same (= num_bins)

#print(len(n))

#print(len(mean_outcome))

#print(len(mean_prediction))

#print(len(numerator))

print("counts:", len(counts), "counts after 0s removed:", len(valid_counts))

print("bins:", len(bin_edges), "bins after 0s removed:", len(valid_bin_edges))

ICI_equivalent = np.sum(numerator) / len(y_test)

return ICI_equivalent

**#calculate ICI equivalent**

print("ICI equivalent: ", ICI_equivalent)

# Appendix 4: Refined definitions of renal disease and primary cancer

Renal disease: ICD-10 codes have demonstrably poor sensitivity for capturing chronic kidney disease (CKD), resulting in underestimation of this highly prevalent condition (Fleet et al., 2013). We therefore sought to leverage the Ontario Laboratory Information System (OLIS) to define CKD as stage 5, 4, 3b, 3a, and 2, defined as having at least two estimated glomerular filtration rates (eGFR) values >90 days apart and less than 1 year apart that was <15, <30, <45, <60, and <90 mL/min, respectively. Higher-stage disease took precedent. eGFR was measured using the 2021 CKD-EPI formula that is not race-adjusted (Lu et al., 2023). Serum creatinine (LOINC codes 14682-9) was only considered if 10-3000 mmol/L. For models that use the definition of CKD using OLIS, we added a comorbidity category for nephritic syndrome (N03, N05) and removed others defined using ICD-10 codes.

Primary cancer: We compared the original cancer definition to one using the Ontario Cancer Registry (OCR), a validated high-quality dataset that scrupulously assigns a cancer diagnosis to a patient based on a laboratory and/or clinical evidence of a diagnosis. The OCR is the gold standard for primary cancer diagnosis in Ontario. Cancers were categorized using the SEER recode classification system, restricting to incident primary malignancies (behaviour code 3). The most recent diagnosis was retained. This was accomplished in two ways. First, the SEER recodes were one-hot encoded. Second, we leveraged one of the strengths of CatBoost: the use of ordered boosting to mitigate target leakage and prediction shift (biases stemming from how categorical variables are encoded when not simply one-hot encoded). With ordered boosting, subsequent trees are trained by incorporating the residuals (Newton gradients) and the ordered target encodings are estimated from prior trees (Kunapuli, 2023). Unlike greedy target encoding, whereby each categorical value assumes a numeric value equal to the mean outcome from the entire training dataset, ordered target encoding randomly shuffles the training dataset (once by default), and the encoding is estimated and updated with each subsequent tree. Ordered target encoding avoids creating a large number of sparse features (e.g., one-hot encoding creates one column per category). The encoding for uncommon categories are smoothed towards the target’s global mean, as would be the encodings encountered in the test set that were not observed in the training data.

# Appendix 5: Features

| **Feature in model** | **Feature names** | **Source** | **Levels** | **Look-back** | **Variable type** |
| --- | --- | --- | --- | --- | --- |
| Age | Age | RPDB | Continuous variable | N/A | Numeric |
| Sex | Sex_M | RPDB | - 1 (male) - 0 (female) | N/A | Binary, already one-hot encoded |
|  |  |  |  |  |  |
| **Various comorbidities from the Charlson comorbidity index** | | | | | |
| Broken out by source | MI, CHF, CVD, PVD, CPD, CTDRD, PUD, MLD, MSLD, DWOC DWC, hemi, dementia, nephritic, cancer, met, MMRD, CKD5 | DAD, NACRS | - Absent - NACRS only - DAD only - Both DAD and NACRS | 3 years | Categorical, subjected to one-hot encoding |
| Included in all models (broken out by source or simple dichotomy) | - met_yn [only when OCR_dx is used] - HIV_1 (HIV); HIV_2 (AIDS) | DAD, NACRS | - Absent (0) - Present (1) | 3 years | Binary, already one-hot encoded |
| Simple dichotomy | MI_yn, CHF_yn, CVD_yn, PVD_yn, CPD_yn, CTDRD_yn, PUD_yn, diabetes_yn_1 (DWOC only), diabetes_yn_2 (DWC), liver_disease_yn_1 (MLD only), liver_disease_yn_2 (MSLD), hemi_yn, dementia_yn, cancer_met_yn_1 (primary cancer only), caner_met_yn_2 (metastatic cancer), MMRD_yn, CKD5_yn | DAD, NACRS | - Absent (0) - Present (1) | 3 years | Binary, already one-hot encoded |
|  |  |  |  |  |  |
| **Comorbidities defined from a different source** | | | | | |
| Primary cancer diagnosis | OCR_dx | Ontario Cancer Registry (OCR) | - No cancer - 76 different cancer types (see eTable S3) | 1 year | Categorical, subjected to one-hot encoding or ordered target statistics encoding (CatBoost sensitivity analysis only) |
| Chronic kidney disease | CKD_stage_OLIS | Ontario Laboratory Information System (OLIS) | - No CKD (eGFR 90+) - Stage 2 (eGFR < 90 mL/min) - Stage 3a (eGFR 45 to <60 mL/min) - Stage 3b (eGFR 30 to <45 mL/min) - Stage 4 (eGFR 15 to <30 mL/min) - Stage 5 (eGFR <15 mL/min) | 3 years | Categorical, subjected to one-hot encoding |
|  |  |  |  |  |  |
| **Other measures of health and healthcare use** | | | | | |
| Various measures of health | OHIP_obese, OHIP_LTC, OHIP_Housecall, OHIP_MH, chronic_pain, fibromyal, palliative, smoking, homecare, substance, criticalcare, hyperbaric, majorburn, amputation, ASA_ASA 3 (ASA III), ASA_ASA 4 (ASA IV), ASA_ASA 5 (ASA V), OHIP_flu, OHIP_BC_screen | Ontario Health Insurance Provider database | - Absent (0) - Present (1) | 1 year | Binary, already one-hot encoded |
| Various measures of health | vaginal_delivery, C_section, delirium, pressure_injury, hip_fracture | DAD | - Absent (0) - Present (1) | 3 years | Binary, already one-hot encoded |
| Activity of Daily Living (ADL) | ADL_score | InterRAI-HC and CCRS | - -99 (no ADL score) - 0 to 28 | 1 year | Integer |
| Counts of healthcare utilization |  |  |  |  |  |
| Counts | num_OHIP | OHIP | Continuous variable | 1 year | Integer |
| Counts | num_DAD | DAD | Continuous variable | 1 year | Integer |
| Counts | num_NACRS, | NACRS | Continuous variable | 1 year | Integer |

# Appendix 6: Python code for training and testing Model 1H

**#Import required packages**

import pandas as pd

from sklearn.model_selection import train_test_split

from sklearn.tree import DecisionTreeClassifier

from sklearn.metrics import accuracy_score, classification_report

from sklearn.metrics import roc_auc_score, roc_curve

import matplotlib.pyplot as plt

import numpy as np

**# For simplicity, this is the list of specific columns included in the model**

X2 = df_train[['Age','Sex_M', 'MI_yn', 'CHF_yn', 'CVD_yn', 'PVD_yn', 'CPD_yn', 'CTDRD_yn', 'PUD_yn', 'diabetes_yn_1', 'diabetes_yn_2', 'liver_disease_yn_1', 'liver_disease_yn_2', 'hemi_yn', 'dementia_yn', 'cancer_met_yn_1', 'cancer_met_yn_2', 'MMRD_yn', 'CKD5_yn', 'HIV_1', 'HIV_2', 'OHIP_obese', 'OHIP_LTC', 'OHIP_Housecall', 'OHIP_MH', 'chronic_pain', 'fibromyal', 'palliative', 'smoking', 'homecare', 'substance', 'criticalcare', 'hyperbaric', 'vaginal_delivery','C_section', 'majorburn', 'amputation', 'delirium', 'pressure_injury', 'hip_fracture', 'num_OHIP', 'num_DAD', 'num_NACRS', 'ASA_ASA 3', 'ASA_ASA 4', 'ASA_ASA 5', 'OHIP_flu', 'OHIP_BC_screen', 'ADL_score' ]]

**# The target variable (dead_1yr) has to be separated from the features in the DataFrame**

Y2 = df_train['dead_1yr']

**# (Optional) First let's delete things from the previous run to ensure errors are caught (rather than recycled from earlier)**

try:

del brier_score_train, brier_score_test, ICI, mean_predicted_value, fraction_of_positives, pr_auc_train, pr_auc_test, auroc_train, auroc_test, X_train, X_test, y_train, y_test

except:

pass

**# Split the data into training and test sets (setting stratify=Y2 to ensure the ratios of the outcome are the same in the train and test sets. This is important for unbalanced datasets)**

X_train, X_test, y_train, y_test = train_test_split(X2, Y2, test_size=0.3, random_state=42, stratify=Y2)

**# Set up the CatBoost classifier model**

from catboost import CatBoostClassifier, Pool

timestamp_start = datetime.now()

meow = CatBoostClassifier(iterations=1000, learning_rate=0.05, verbose=0, min_data_in_leaf=10, depth=6)

meow.fit(X_train, y_train)

timestamp_end = datetime.now()

**# Predict class labels on the test set**

y_pred_prob_train = meow.predict_proba(X_train)[:, 1] # Probabilities for class 1 in the training set (necessary because a 1-D array is needed)

y_pred_prob_test = meow.predict_proba(X_test)[:, 1] # Probabilities for class 1 in the test set

**# Calculate AUROC**

auroc_train = roc_auc_score(y_train, y_pred_prob_train)

auroc_test = roc_auc_score(y_test, y_pred_prob_test)

print("AUROC train:", auroc_train)

print("AUROC test:", auroc_test)

**# Calculate PR-AUC**

pr_auc_train = average_precision_score(y_train, y_pred_prob_train)

pr_auc_test = average_precision_score(y_test, y_pred_prob_test)

print("PR-AUC train:", pr_auc_train)

print("PR-AUC test:", pr_auc_test)

**# Calculate Brier Score for training and test sets**

brier_score_train = brier_score_loss(y_train, y_pred_prob_train)

brier_score_test = brier_score_loss(y_test, y_pred_prob_test)

print("Brier Score (Train):", brier_score_train)

print("Brier Score (Test):", brier_score_test)

**# Calculate the ROC curve (FPR, TPR, and thresholds)**

fpr, tpr, thresholds = roc_curve(y_test, y_pred_prob_test)

**# Ensure y_test and y_pred_prob_test are numpy arrays**

y_test = np.array(y_test)

y_pred_prob_test = np.array(y_pred_prob_test)

**# Calculate run time**

minutes = (timestamp_end - timestamp_start).total_seconds() / 60

hours = (timestamp_end - timestamp_start).total_seconds() / 60 / 60

print("Minutes:", minutes)

print("Hours:", hours)

**#Calculate ICI equivalent [see Appendix 1 for this function]**

ICI_equivalent = calculate_ici_equivalent(y_pred_prob_test, y_test, num_bins=100)

print("ICI equivalent", ICI_equivalent)

**#Calibration curve using centiles (100 bins)**

from sklearn.calibration import calibration_curve

fraction_of_positives, mean_predicted_value = calibration_curve(y_test, y_pred_prob_test, n_bins=100)

**# Create a figure and two subplots (1 row, 2 columns)**

fig, ax = plt.subplots(1, 2, figsize=(12, 5)) # 1 row, 2 columns

**# First plot: Calibration curve**

ax[0].plot(mean_predicted_value, fraction_of_positives, marker='o', label=f'Calibration Curve')

ax[0].plot([0, 1], [0, 1], linestyle='--', label='Perfectly calibrated')

ax[0].set_title('Calibration Curve')

ax[0].set_xlabel('Mean Predicted Probability')

ax[0].set_ylabel('Fraction of Positives')

ax[0].legend()

# Second plot: ROC curve

ax[1].plot(fpr, tpr, color='blue', label=f'ROC curve (AUROC = {auroc_test:.3f})')

ax[1].plot([0, 1], [0, 1], color='gray', linestyle='--') # Diagonal line (random classifier)

ax[1].set_xlabel('False Positive Rate')

ax[1].set_ylabel('True Positive Rate')

ax[1].set_title('Receiver Operating Characteristic (ROC) Curve')

ax[1].legend(loc='lower right')

ax[1].grid(True)

plt.tight_layout()

plt.show()

**#Distribution of predicted probabilities**

plt.hist(y_pred_prob_test, bins=100)

plt.title("Histogram of Predicted Probabilities")

plt.xlabel("Predicted Probability")

plt.ylabel("Frequency")

plt.show()

**# Note: the mean predicted probability is very close to the mean outcome**

print(y_pred_prob_test.mean())

print(y_test.mean())

**#the range is quite wide too**

print(f"Range of y_pred_prob_test: {y_pred_prob_test.min()} to {y_pred_prob_test.max()}")

# Appendix 7: Prevalence and Crude Risk of 1-Year All-Cause Mortality by cancer type

| **Cancer Diagnosis (OCR)** | **Total** | **Prevalence** | **N dead** | **% dead** |
| --- | --- | --- | --- | --- |
| No cancer | 11,912,425 | 98.6 | 105,941 | 0.89 |
| Breast | 30,868 | 0.26 | 1,047 | 3.39 |
| Prostate | 22,590 | 0.19 | 958 | 4.24 |
| Lung, Bronchus, other | 13,808 | 0.11 | 3,248 | 23.5 |
| Melanoma of the Skin | 9,865 | 0.08 | 520 | 5.27 |
| Corpus Uteri | 7,699 | 0.06 | 382 | 4.96 |
| Thyroid | 7,382 | 0.06 | 75 | 1.02 |
| Kidney and Renal Pelvis | 6,175 | 0.05 | 366 | 5.93 |
| Rectum | 4,725 | 0.04 | 495 | 10.5 |
| NHL - Nodal | 4,633 | 0.04 | 411 | 8.87 |
| NHL - Extranodal | 4,629 | 0.04 | 395 | 8.53 |
| Urinary Bladder | 4,561 | 0.04 | 619 | 13.6 |
| Sigmoid Colon | 3,143 | 0.03 | 285 | 9.07 |
| Myeloma | 3,007 | 0.02 | 353 | 11.7 |
| Ovary | 2,504 | 0.02 | 367 | 14.7 |
| Ascending Colon | 2,471 | 0.02 | 263 | 10.6 |
| Cecum | 2,419 | 0.02 | 266 | 11.0 |
| Stomach | 2,350 | 0.02 | 519 | 22.1 |
| Pancreas | 2,207 | 0.02 | 848 | 38.4 |
| Chronic Lymphocytic Leukemia | 2,195 | 0.02 | 113 | 5.15 |
| Tongue | 1,579 | 0.01 | 159 | 10.1 |
| Cervix Uteri | 1,432 | 0.01 | 79 | 5.52 |
| Liver | 1,325 | 0.01 | 334 | 25.2 |
| Brain | 1,317 | 0.01 | 473 | 35.9 |
| Rectosigmoid Junction | 1,245 | 0.01 | 132 | 10.6 |
| Testis | 1,226 | 0.01 | 12 | 0.98 |
| Soft Tissue including Heart | 1,195 | 0.01 | 133 | 11.1 |
| Transverse Colon | 1,117 | 0.01 | 123 | 11.0 |
| Hodgkin lymphoma | 1,093 | 0.01 | 24 | 2.20 |
| Small Intestine | 1,090 | 0.01 | 125 | 11.5 |
| Esophagus | 995 | 0.01 | 332 | 33.4 |
| Miscellaneous | 976 | 0.01 | 205 | 21.0 |
| Other Urinary Organs | 894 | 0.01 | 162 | 18.1 |
| Other Non-Epithelial Skin | 868 | 0.01 | 96 | 11.1 |
| Acute Myeloid Leukemia | 865 | 0.01 | 240 | 27.7 |
| Larynx | 833 | 0.01 | 100 | 12.0 |
| Anus, Anal Canal and Anorectum | 809 | 0.01 | 93 | 11.5 |
| Chronic Myeloid Leukemia | 797 | 0.01 | 86 | 10.8 |
| Descending Colon | 792 | 0.01 | 61 | 7.70 |
| Appendix | 764 | 0.01 | 33 | 4.32 |
| Tonsil | 762 | 0.01 | 73 | 9.58 |
| Vulva | 744 | 0.01 | 78 | 10.5 |
| Gum and Other Mouth | 722 | 0.01 | 106 | 14.7 |
| Hepatic Flexure | 645 | 0.01 | 69 | 10.7 |
| Other Biliary | 645 | 0.01 | 223 | 34.6 |
| Salivary Gland | 554 | 0.00 | 52 | 9.39 |
| Other Endocrine including Thymus | 454 | 0.00 | 26 | 5.73 |
| Splenic Flexure | 417 | 0.00 | 36 | 8.63 |
| Eye and Orbit | 405 | 0.00 | 20 | 4.94 |
| Intrahepatic Bile Duct | 337 | 0.00 | 149 | 44.2 |
| Nose, Nasal Cavity and Middle Ear | 275 | 0.00 | 23 | 8.36 |
| Bones and Joints | 270 | 0.00 | 39 | 14.4 |
| Lip | 269 | 0.00 | 25 | 9.29 |
| Nasopharynx | 261 | 0.00 | 16 | 6.13 |
| Gallbladder | 253 | 0.00 | 74 | 29.2 |
| Oropharynx | 240 | 0.00 | 31 | 12.9 |
| Acute Lymphocytic Leukemia | 230 | 0.00 | 28 | 12.2 |
| Other Lymphocytic Leukemia | 222 | 0.00 | 14 | 6.31 |
| Floor of Mouth | 218 | 0.00 | 24 | 11.0 |
| Mesothelioma | 200 | 0.00 | 84 | 42.0 |
| Penis | 198 | 0.00 | 21 | 10.6 |
| Other Female Genital Organs | 185 | 0.00 | 21 | 11.4 |
| Vagina | 158 | 0.00 | 22 | 13.9 |
| Retroperitoneum | 156 | 0.00 | 20 | 12.8 |
| Large Intestine, NOS | 133 | 0.00 | 31 | 23.3 |
| Other Oral Cavity and Pharynx | 116 | 0.00 | 19 | 16.4 |
| Hypopharynx | 109 | 0.00 | 29 | 26.6 |
| Cranial Nerves Other Nervous System | 107 | 0.00 | 10 | 9.35 |
| Aleukemic, subleukemic and NOS | 96 | 0.00 | 11 | 11.5 |
| Kaposi Sarcoma | 94 | 0.00 | 5 | 5.32 |
| Other Digestive Organs | 79 | 0.00 | 25 | 31.6 |
| Ureter | 79 | 0.00 | 13 | 16.5 |
| Other Male Genital Organs | 70 | 0.00 | 7 | 10.0 |
| Unknown | 67 | 0.00 | 18 | 26.9 |
| Other leukemia | 63 | 0.00 | 15 | 23.8 |
| Uterus, NOS | 51 | 0.00 | 11 | 21.6 |
| Peritoneum, Omentum and Mesentery | 49 | 0.00 | 10 | 20.4 |

*NOS – not otherwise specified

# Appendix 8: Predictors of 1-year mortality from logistic regression, adjusted for all variables shown

|  | **Log odds** | **Standard error** | **p-value** | **OR (95% CI)** | **p-value*** |
| --- | --- | --- | --- | --- | --- |
| Constant | -10.1397 | 0.024 | 0 |  |  |
| Age (per 10 years) | 0.0769 | 0 | 0 | 2.16 (2.14-2.16) |  |
| Sex (male) | 0.2932 | 0.008 | 0 | 1.34 (1.32-1.36) |  |
|  |  |  |  |  |  |
| Myocardial infarction | -0.0175 | 0.022 | 0.433 | 0.98 (0.94-1.03) | ns |
| Congestive heart failure | 0.5462 | 0.015 | 0 | 1.73 (1.68-1.78) |  |
| Cerebrovascular disease | 0.0552 | 0.018 | 0.002 | 1.06 (1.02-1.10) |  |
| Peripheral vascular disease | 0.2666 | 0.024 | 0 | 1.31 (1.24-1.37) |  |
| Cardiopulmonary disease | 0.6655 | 0.015 | 0 | 1.95 (1.89-2.00) |  |
| Connective tissue / rheumatoid disease | 0.3226 | 0.045 | 0 | 1.38 (1.26-1.51) |  |
| Peptic ulcer disease | 0.1648 | 0.031 | 0 | 1.18 (1.11-1.25) |  |
|  |  |  |  |  |  |
| Diabetes (no complications) | 0.1866 | 0.015 | 0 | 1.21 (1.17-1.24) |  |
| Diabetes (with complications) | 0.2585 | 0.013 | 0 | 1.29 (1.26-1.33) |  |
| Liver disease (mild) | 0.7931 | 0.035 | 0 | 2.21 (2.06-2.37) |  |
| Liver disease (moderate/severe) | 1.7359 | 0.037 | 0 | 5.67 (5.28-6.09) |  |
|  |  |  |  |  |  |
| Hemiplegia/paraplegia | 0.0381 | 0.042 | 0.364 | 1.04 (0.96-1.13) | ns |
| Dementia | 0.2709 | 0.018 | 0 | 1.31 (1.27-1.36) |  |
| Metastatic cancer | 1.3082 | 0.021 | 0 | 3.70 (3.55-3.85) |  |
|  |  |  |  |  |  |
| HIV | 0.8005 | 0.174 | 0 | 2.23 (1.58-3.13) |  |
| AIDS | 0.3934 | 0.272 | 0.147 | 1.48 (0.87-2.52) | ns |
|  |  |  |  |  |  |
| Nephritic syndrome | 0.0089 | 0.134 | 0.947 | 1.01 (0.77-1.31) | ns |
| Chronic Kidney Disease (CKD-EPI) |  |  |  |  |  |
| Stage 2 (eGFR < 90 mL/min) | 0.0147 | 0.01 | 0.146 | 1.01 (1.00-1.03) | ns |
| Stage 3a (eGFR 45 to <60 mL/min) | 0.2014 | 0.014 | 0 | 1.22 (1.19-1.26) |  |
| Stage 3b (eGFR 30 to <45 mL/min) | 0.4029 | 0.017 | 0 | 1.50 (1.45-1.55) |  |
| Stage 4 (eGFR 15 to <30 mL/min) | 0.8842 | 0.024 | 0 | 2.42 (2.31-2.54) |  |
| Stage 5 (eGFR <15 mL/min) | 0.7625 | 0.047 | 0 | 2.14 (1.95-2.35) |  |
|  |  |  |  |  |  |
| Primary cancer (Ontario Cancer Registry) |  |  |  |  |  |
| Acute Lymphocytic Leukemia | 1.4378 | 0.315 | 0 | 4.21 (2.27-7.80) |  |
| Acute Myeloid Leukemia | 2.1974 | 0.117 | 0 | 9.00 (7.16-11.32) |  |
| Aleukemic, subleukemic and NOS | 0.794 | 0.53 | 0.134 | 2.21 (0.78-6.25) | ns |
| Anus, Anal Canal and Anorectum | 1.3722 | 0.15 | 0 | 3.94 (2.94-5.29) |  |
| Appendix | 0.4817 | 0.247 | 0.051 | 1.62 (1.00-2.63) | ns |
| Ascending Colon | 0.3698 | 0.092 | 0 | 1.45 (1.21-1.73) |  |
| Bones and Joints | 1.9705 | 0.253 | 0 | 7.17 (4.37-11.78) |  |
| Brain | 3.845 | 0.089 | 0 | 46.76 (39.25-55.70) |  |
| Breast | 0.1658 | 0.045 | 0 | 1.18 (1.08-1.29) |  |
| Cecum | 0.3039 | 0.097 | 0.002 | 1.36 (1.12-1.64) |  |
| Cervix Uteri | 1.7183 | 0.166 | 0 | 5.58 (4.03-7.72) |  |
| Chronic Lymphocytic Leukemia | 0.5668 | 0.126 | 0 | 1.76 (1.38-2.25) |  |
| Chronic Myeloid Leukemia | 1.2177 | 0.155 | 0 | 3.38 (2.49-4.58) |  |
| Corpus Uteri | 1.1209 | 0.072 | 0 | 3.07 (2.66-3.53) |  |
| Cranial Nerves Other Nervous System | 0.3384 | 0.63 | 0.591 | 1.40 (0.41-4.82) | ns |
| Descending Colon | 0.2794 | 0.187 | 0.136 | 1.32 (0.92-1.91) | ns |
| Esophagus | 2.0133 | 0.098 | 0 | 7.49 (6.18-9.08) |  |
| Eye and Orbit | 0.3118 | 0.342 | 0.363 | 1.37 (0.70-2.67) | ns |
| Floor of Mouth | 1.2785 | 0.292 | 0 | 3.59 (2.03-6.37) |  |
| Gallbladder | 2.0788 | 0.191 | 0 | 7.99 (5.50-11.63) |  |
| Gum and Other Mouth | 1.3941 | 0.153 | 0 | 4.03 (2.99-5.44) |  |
| Hepatic Flexure | 0.4133 | 0.178 | 0.02 | 1.51 (1.07-2.14) |  |
| Hodgkin lymphoma | 0.4785 | 0.277 | 0.084 | 1.61 (0.94-2.78) | ns |
| Hypopharynx | 1.7835 | 0.314 | 0 | 5.95 (3.21-11.02) |  |
| Intrahepatic Bile Duct | 2.356 | 0.172 | 0 | 10.55 (7.54-14.76) |  |
| Kaposi Sarcoma | 0.3393 | 0.563 | 0.546 | 1.40 (0.47-4.23) | ns |
| Kidney and Renal Pelvis | 0.3641 | 0.074 | 0 | 1.44 (1.24-1.67) |  |
| Large Intestine, NOS | 1.0939 | 0.326 | 0.001 | 2.99 (1.58-5.65) |  |
| Larynx | 1.0428 | 0.151 | 0 | 2.84 (2.11-3.82) |  |
| Lip | 0.9572 | 0.293 | 0.001 | 2.60 (1.47-4.62) |  |
| Liver | 1.0899 | 0.095 | 0 | 2.97 (2.47-3.58) |  |
| Lung, Bronchus, other | 1.3348 | 0.032 | 0 | 3.80 (3.57-4.04) |  |
| Melanoma of the Skin | 0.3717 | 0.062 | 0 | 1.45 (1.28-1.64) |  |
| Mesothelioma | 2.3875 | 0.202 | 0 | 10.89 (7.32-16.18) |  |
| Miscellaneous | 1.1554 | 0.12 | 0 | 3.18 (2.51-4.02) |  |
| Myeloma | 0.4482 | 0.079 | 0 | 1.57 (1.34-1.83) |  |
| NHL - Extranodal | 0.763 | 0.075 | 0 | 2.14 (1.85-2.48) |  |
| NHL - Nodal | 0.9415 | 0.073 | 0 | 2.56 (2.22-2.96) |  |
| Nasopharynx | 1.019 | 0.327 | 0.002 | 2.77 (1.46-5.26) |  |
| Nose, Nasal Cavity and Middle Ear | 1.1696 | 0.297 | 0 | 3.22 (1.80-5.77) |  |
| Oropharynx | 1.6812 | 0.239 | 0 | 5.37 (3.36-8.58) |  |
| Other Biliary | 1.8992 | 0.124 | 0 | 6.68 (5.24-8.52) |  |
| Other Digestive Organs | 1.9267 | 0.344 | 0 | 6.87 (3.50-13.48) |  |
| Other Endocrine including Thymus | 0.6903 | 0.301 | 0.022 | 1.99 (1.10-3.60) |  |
| Other Female Genital Organs | 1.0878 | 0.322 | 0.001 | 2.97 (1.58-5.58) |  |
| Other Lymphocytic Leukemia | 0.9664 | 0.361 | 0.007 | 2.63 (1.30-5.33) |  |
| Other Male Genital Organs | 0.3773 | 0.582 | 0.517 | 1.46 (0.47-4.56) | ns |
| Other Non-Epithelial Skin | 0.7236 | 0.152 | 0 | 2.06 (1.53-2.78) |  |
| Other Oral Cavity and Pharynx | 1.3503 | 0.347 | 0 | 3.86 (1.96-7.61) |  |
| Other Urinary Organs | 1.0387 | 0.123 | 0 | 2.83 (2.22-3.59) |  |
| Other leukemia | 1.6858 | 0.568 | 0.003 | 5.40 (1.77-16.43) |  |
| Ovary | 1.5251 | 0.085 | 0 | 4.60 (3.89-5.42) |  |
| Pancreas | 2.2175 | 0.065 | 0 | 9.18 (8.08-10.43) |  |
| Penis | 0.1131 | 0.358 | 0.752 | 1.12 (0.55-2.26) | ns |
| Peritoneum, Omentum and Mesentery | 0.5064 | 0.607 | 0.405 | 1.66 (0.50-5.46) | ns |
| Prostate | -0.0782 | 0.046 | 0.09 | 0.92 (0.84-1.01) | ns |
| Rectosigmoid Junction | 0.6847 | 0.13 | 0 | 1.98 (1.54-2.56) |  |
| Rectum | 0.7653 | 0.069 | 0 | 2.15 (1.88-2.46) |  |
| Retroperitoneum | 1.4159 | 0.331 | 0 | 4.12 (2.15-7.89) |  |
| Salivary Gland | 0.6005 | 0.197 | 0.002 | 1.82 (1.24-2.68) |  |
| Sigmoid Colon | 0.5695 | 0.088 | 0 | 1.77 (1.49-2.10) |  |
| Small Intestine | 0.6943 | 0.136 | 0 | 2.00 (1.53-2.62) |  |
| Soft Tissue including Heart | 1.2274 | 0.139 | 0 | 3.41 (2.60-4.48) |  |
| Splenic Flexure | 0.4741 | 0.226 | 0.036 | 1.61 (1.03-2.50) |  |
| Stomach | 1.4635 | 0.074 | 0 | 4.32 (3.74-4.99) |  |
| Testis | 0.2854 | 0.414 | 0.491 | 1.33 (0.59-3.00) | ns |
| Thyroid | -0.143 | 0.147 | 0.329 | 0.87 (0.65-1.15) | ns |
| Tongue | 1.0459 | 0.118 | 0 | 2.85 (2.26-3.59) |  |
| Tonsil | 1.2281 | 0.171 | 0 | 3.41 (2.44-4.77) |  |
| Transverse Colon | 0.4459 | 0.14 | 0.001 | 1.56 (1.19-2.05) |  |
| Unknown | 2.3707 | 0.441 | 0 | 10.70 (4.51-25.41) |  |
| Ureter | 0.1968 | 0.518 | 0.704 | 1.22 (0.44-3.36) | ns |
| Urinary Bladder | 0.9229 | 0.062 | 0 | 2.52 (2.23-2.84) |  |
| Uterus, NOS | 3.3452 | 0.492 | 0 | 28.37 (10.80-74.44) |  |
| Vagina | 1.6776 | 0.327 | 0 | 5.35 (2.82-10.16) |  |
| Vulva | 1.2262 | 0.163 | 0 | 3.41 (2.48-4.69) |  |
|  |  |  |  |  |  |
| Obesity | -0.0284 | 0.044 | 0.52 | 0.97 (0.89-1.06) | ns |
| Smoking cessation | 0.4751 | 0.036 | 0 | 1.61 (1.50-1.73) |  |
| Substance abuse | 1.4623 | 0.037 | 0 | 4.32 (4.01-4.64) |  |
| Chronic pain | 0.3772 | 0.2 | 0.059 | 1.46 (0.99-2.16) | ns |
| Fibromyalgia | 0.0227 | 0.061 | 0.709 | 1.02 (0.91-1.15) | ns |
|  |  |  |  |  |  |
| Palliative care | 1.2244 | 0.016 | 0 | 3.40 (3.30-3.51) |  |
| Long-term care | 0.1385 | 0.019 | 0 | 1.15 (1.11-1.19) |  |
| Housecall | -0.0745 | 0.033 | 0.024 | 0.93 (0.87-0.99) | p |
| Mental health | 0.2627 | 0.025 | 0 | 1.30 (1.24-1.37) |  |
| Homecare | 0.425 | 0.014 | 0 | 1.53 (1.49-1.57) |  |
|  |  |  |  |  |  |
| Critical care | 0.3395 | 0.013 | 0 | 1.40 (1.37-1.44) |  |
| Hyperbaric treatment | 0.2088 | 0.167 | 0.21 | 1.23 (0.89-1.71) | ns |
| Major burn | -0.6675 | 0.636 | 0.294 | 0.51 (0.15-1.79) | ns |
| Amputation | 0.1859 | 0.079 | 0.018 | 1.20 (1.03-1.40) |  |
|  |  |  |  |  |  |
| Delirium | -0.0087 | 0.017 | 0.619 | 0.99 (0.96-1.03) | ns |
| Pressure injury | 0.1714 | 0.062 | 0.006 | 1.19 (1.05-1.34) |  |
| Hip fracture | 0.0207 | 0.024 | 0.389 | 1.02 (0.97-1.07) | ns |
|  |  |  |  |  |  |
| OHIP count | 0.0061 | 0 | 0 | 1.01 (1.01-1.01) |  |
| Hospitalization count | 0.0353 | 0.006 | 0 | 1.04 (1.02-1.05) |  |
| Hospital ambulatory visit count | 0.0053 | 0 | 0 | 1.01 (1.01-1.01) |  |
|  |  |  |  |  |  |
| ASA score 3 | -0.2375 | 0.018 | 0 | 0.79 (0.76-0.82) | p |
| ASA score 4 | -0.0683 | 0.017 | 0 | 0.93 (0.90-0.97) | p |
| ASA score 5 | -0.0059 | 0.064 | 0.927 | 0.99 (0.88-1.13) | ns |
|  |  |  |  |  |  |
| Vaginal delivery | -0.5555 | 0.131 | 0 | 0.57 (0.44-0.74) | p |
| Caesarean section delivery | -0.7779 | 0.201 | 0 | 0.46 (0.31-0.68) | p |
| Influenza vaccine | -0.1857 | 0.011 | 0 | 0.83 (0.81-0.85) | p |
| Breast cancer screening (mammography) | -0.6474 | 0.016 | 0 | 0.52 (0.51-0.54) | p |
| Activities of Daily Living score (per point) | 0.0773 | 0.001 | 0 | 1.08 (1.08-1.08) |  |
| * summary p-value (p=protective, ns=not significant at alpha=0.05, and deleterious if blank)  eGFR – estimated glomerular filtration rate; ASA | | | | | |

# Appendix 9: Marginal effects from Model 1H

|  | Marginal effect^a^ | |
| --- | --- | --- |
|  | Average | Relative |
| Palliative care | 4.03% | 4.38 |
| Moderate/severe liver disease | 2.60% | 2.61 |
| Metastatic cancer | 1.53% | 1.59 |
| Pressure injury | 1.20% | 1.19 |
| Congestive heart failure | 0.97% | 1.00 |
| Dementia | 0.96% | 0.96 |
| Mile liver disease | 0.83% | 0.83 |
| ASA 5 | 0.82% | 0.81 |
| Cardiopulmonary disease | 0.73% | 0.75 |
| Activities of Daily Living (per point) | 0.66% | 0.66 |
| Substance abuse | 0.63% | 0.63 |
| Homecare | 0.51% | 0.52 |
| Delirium | 0.51% | 0.51 |
| Mild-to-moderate renal disease | 0.51% | 0.51 |
| Primary cancer | 0.49% | 0.51 |
| Diabetes with complications | 0.42% | 0.43 |
| Long-term care residence | 0.42% | 0.42 |
| Amputation | 0.41% | 0.41 |
| Hip fracture | 0.35% | 0.34 |
| Smoking cessation | 0.34% | 0.34 |
| Critical care | 0.34% | 0.34 |
| Severe renal disease (stage 5, CIHI) | 0.31% | 0.31 |
| Peripheral vascular disease | 0.31% | 0.31 |
| Sex (male) | 0.30% | 0.34 |
| Connective tissue / rheumatoid disease | 0.22% | 0.22 |
| Number of admissions (per additional visit) | 0.16% | 0.16 |
| Diabetes without complications | 0.13% | 0.13 |
| Number of outpatient hospital visits (per additional visit) | 0.11% | 0.11 |
| Mental health | 0.09% | 0.09 |
| Peptic ulcer disease | 0.09% | 0.09 |
| Hemiplegia/paraplegia | 0.08% | 0.08 |
| ASA 4 | 0.08% | 0.07 |
| HIV | 0.07% | 0.07 |
| Age (per year) | 0.06% | 0.06 |
| Cerebrovascular disease | 0.06% | 0.06 |
| Number of healthcare visits (per additional visit) | 0.01% | 0.01 |
| Caesarean section delivery | 0.00% | 0.00 |
| Major burn | 0.00% | 0.00 |
| Housecall | -0.01% | -0.01 |
| Myocardial infarction | -0.01% | -0.01 |
| Fibromyalgia | -0.03% | -0.03 |
| Obesity | -0.10% | -0.10 |
| Hyperbaric treatment | -0.10% | -0.10 |
| Vaginal delivery | -0.12% | -0.12 |
| Chronic pain | -0.14% | -0.14 |
| Influenza vaccination | -0.14% | -0.13 |
| AIDS | -0.21% | -0.20 |
| ASA 3 | -0.24% | -0.24 |
| Breast cancer screening | -0.29% | -0.28 |
| 95% confidence intervals for the marginal effects are close to the point estimate due to sample size, so are not shown for clarity | | |
